# Reproducibility and Repeatability of Computer Tomography-based Measurement of Abdominal Subcutaneous and Visceral Adipose Tissues

**DOI:** 10.1038/srep40389

**Published:** 2017-01-10

**Authors:** Yuan-Hao Lee, Hsing-Fen Hsiao, Hou-Ting Yang, Shih-Yi Huang, Wing P. Chan

**Affiliations:** 1 Department of Radiology, Wan Fang Hospital, Taipei Medical University, Taipei 116, Taiwan, Republic of China; 2 Department of Nuclear Medicine, Chang Gung Memorial Hospital, Taoyuan, Taiwan, Republic of China; 3 Program of Electrical and Communications Engineering, Feng Chia University, Taichung, Taiwan, Republic of China; 4 School of Nutrition and Health Sciences, Taipei Medical University, Taipei 110, Taiwan, Republic of China; 5 Department of Radiology, School of Medicine, College of Medicine, Taipei Medical University, Taipei 110, Taiwan, Republic of China

## Abstract

Excessive accumulation of abdominal adipose tissue is a widely recognized as a major feature of obesity, and it can be quantified by dual-energy x-ray absorptiometry (DXA). However, in a phantom study, the inter- and intra-instrument reliability of DXA remains unpredictable. Thus, we attempted to determine the precision of estimates from computer tomography-based measurements and analysis with AZE Virtual Place software. To determine the inter-rater reproducibility and intra-rater repeatability of adipose tissue area estimates, we used the automatic boundary-tracing function of the AZE Virtual Place to generate cross-sectional areas of subcutaneous and visceral adipose tissues from the abdomen of reconstructed CT images. The variability of inter-rater and intra-rater estimates expressed as the coefficient of variation ranged from 0.47% to 1.43% for subcutaneous adipose tissue and 1.08% to 2.20% for visceral adipose tissue; the optimal coefficient of variation of the fat rate calculation ranged from 0.55% to 1.13%, respectively. There was high and significant correlation between adipose tissue areas as estimated in 40 obese subjects by two raters or repeatedly on 20 obese subjects by either rater. This indicates excellent reproducibility and repeatability via a computer tomography-based measurement of abdominal subcutaneous and visceral adipose tissues.

Obesity is defined as abnormal or excessive fat accumulation and is associated with many health and social problems including cardiovascular diseases, type 2 diabetes, and disability^1^,^2^. Obese subjects with visceral fat accumulation have greater impairment of glucose and lipid metabolism than those with subcutaneous fat accumulation^3^,^4^,^5^. Visceral fat accumulation and metabolic syndrome including glucose intolerance, hyperlipidemia, and hypertension are also correlated in non-obese subjects^6^,^7^,^8^. A reduction in abdominal visceral fat obesity and increase skeletal muscle mass can improve the quality of life and lower the severity of metabolic syndrome^9^,^10^,^11^. Thus, finding a good method to analyze and monitor the effect of treatment on abdominal and visceral adiposity is important.

Currently, intra-abdominal adipose tissue is measured using computed tomography (CT), magnetic resonance imaging (MRI), ultrasound, and dual energy absorptiometry (DXA)^12^,^13^,^14^,^15^. CT is an optimal tool for relatively rapid and accurate measurement of adipose tissue with low radiation exposure^12^,^16^,^17^. MRI has been used less frequently because of lower access and higher cost^17^. Ultrasonography provides a reliable estimate of subcutaneous fat thickness, but is an imprecise estimate of adipose tissue volume^3^. The CoreScan use of DXA can estimate visceral adipose tissue (VAT) content^18^. However, the inter- and intra- instrument variability of CoreScan estimates is high^19^. This limits the clinical utility of the findings including applications such as fat removal or cosmetic surgery^18^.

Through studies with CT scans, the Examination Committee of Criteria for Obesity Disease in Japan suggested that the optimal threshold for characterizing individuals at risk for obesity-related disorders was a visceral fat area (of 100 cm^2^)^20^. Unfortunately, VAT measurement on CT has been limited to a single slice due to radiation exposure and cost considerations. These single-slice techniques are often less accurate than VAT volumetric analysis^16^,^17^. Moreover, adipocyte metabolism is differently regulated in women versus men in association with the effect of sex steroid hormones as well as the local density of their specific receptors^21^,^22^. To foster precise assessment of visceral obesity and gender disparities in obesity, increased voxel thickness of CT images are needed to estimate both the VAT and the ratio of VAT to the sum of subcutaneous adipose tissue (SAT) and VAT. A lower baseline visceral-to-abdominal fat ratio is associated with improved metabolic parameters^23^.

Thaete *et al*.^24^ first examined the reproducibility of abdominal adipose tissue with CT scans by repositioning volunteers onto the table for repeat scan. The boundary of SAT and VAT was separated manually using a cursor. Then, other studies revealed that areas of abdominal SAT and VAT can be easily determined via post-processing software such as AZE Virtual Place and Image J^25^,^26^. These offer automated segmentation and manual tracing. The assessment of abdominal SAT and VAT areas with Image J software has been shown to have good inter-observer reproducibility^26^. However, there is a lack of intra-observer repeatability data. Furthermore, VAT is obtained by subtraction of the total fat and SAT without removing the fat-containing tissues from the bowel wall and back muscle within the contoured VAT areas^26^.

In this study, we evaluated the inter-rater reproducibility and intra-rater repeatability of CT-based assessment of abdominal SAT and VAT via AZE Virtual Place. To the best of our knowledge, this is the first report to determine the measurement consistency of SAT and VAT using CT images with AZE Virtual Place.

## Results

### Baseline characteristics

We used 40 subjects to assess inter-rater reproducibility, and 20 out of the 40 subjects were used for intra-rater repeatability. Both assessments had 90% of participants with body mass indices (BMIs) higher than or equal to 25 kg/m^2^ (Table 1 and Supplementary Table 1). Only 8 (20%) and 5 (25%) participants were males, respectively. The average effective dose was 0.166 mSv for all CT scans.

### Inter-rater reproducibility

**Figure 1:**
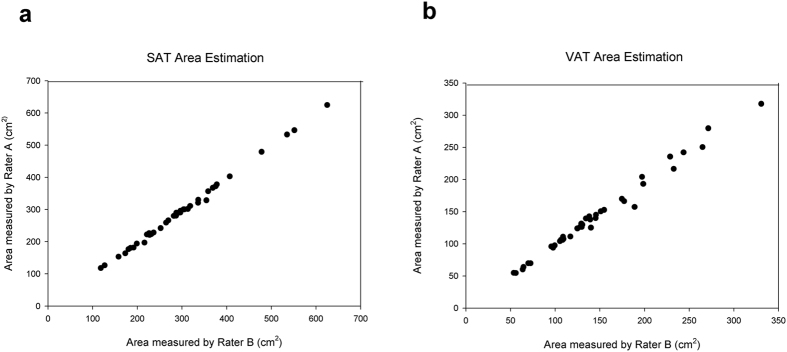
Inter-rater correlation of SAT and VAT area estimates. (**a**) The strength of correlation between the SAT areas measured by Rater A and Rater B was described by Pearson’s correlation coefficient (*r* = 0.99877) and p value (p < 0.0001). (**b**) The inter-rater reproducibility of VAT area estimation was validated using Pearson’s product-moment correlation (*r* = 0.99363, p < 0.0001).

Semi-automated estimation of SAT and VAT areas by AZE Virtual Place took approximately 20 minutes to complete including both CT scanning and image post-processing. Quantitative SAT and VAT estimates along with biological information for 40 subjects are summarized in Table 2. The inter-rater CVs were 1.43% and 2.20% for SAT and VAT area estimates, respectively. The fat rate (the VAT area presented as a percentage of the total [i.e., the sum of SAT and VAT areas]) was also evaluated. The inter-rater CV was smaller for the fat rate (0.96%) than for the SAT and VAT area estimates. The Pearson’s inter-rater correlation coefficients (r) were 0.99877 for SAT area measurements and 0.99363 for VAT area measurements (Fig. 1).

### Intra-rater repeatability

**Figure 2:**
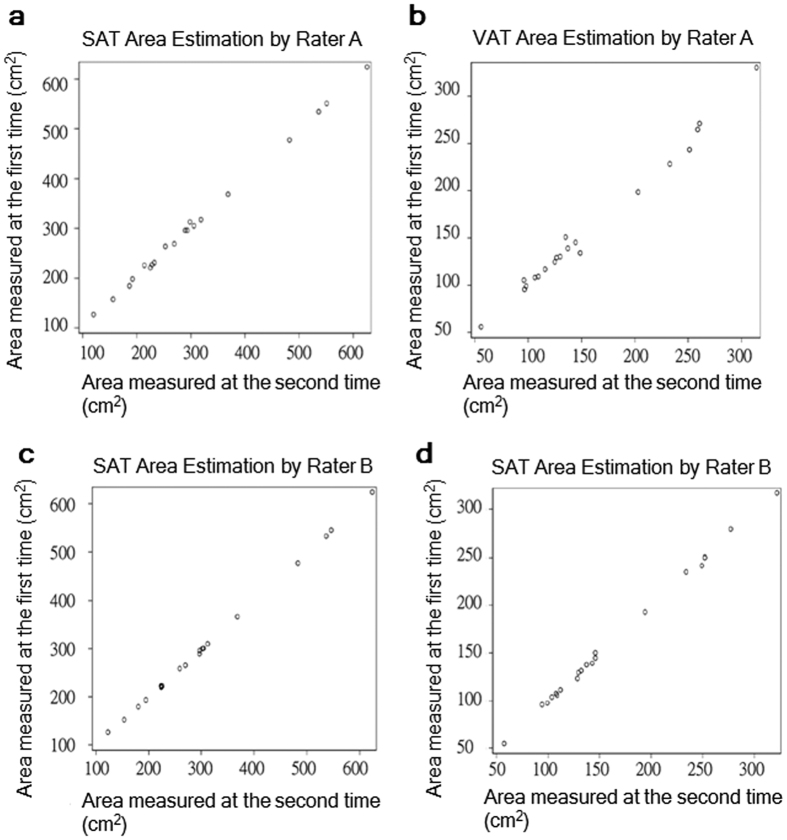
Intra-rater correlation of SAT area and VAT area estimates. The strength of correlation between the 1^st^ and 2^nd^ estimates of SAT or VAT area was validated by Pearson’s correlation coefficient (r) and p value. (**a**) The intra-rater correlations for SAT (r = 0.99930, p < 0.0001) and (**b**) VAT (r = 0.99509, p < 0.0001) area estimates by Rater A. (**c**) The intra-rater correlations for SAT (r = 0.99988, p < 0.0001) and (**b**) VAT (r = 0.99928, p < 0.0001) area estimates by Rater B.

The same CT scans were performed on 20 out of the 40 subjects and were utilized for intra-rater repeatability assessment (demographic information is listed in Supplementary Table 1). The intra-rater CV values were 1.16% and 0.47%, respectively, for SAT area estimation. This was performed twice by Rater A and Rater B. The CV values for VAT area were 2.13% and 1.08% for Rater A and Rater B, respectively (Table 3). The intra-rater repeatability was slightly higher for SAT area estimates (correlation coefficients of Rater A, 0.99930 vs. Rater B, 0.99988) than VAT area estimates (A, 0.99509 vs. B, 0.99928) (Fig. 2). Finally, the intra-rater variation in fat rates was smaller for Rater B (CV of Rater A vs. CV of Rater B, 1.13 vs. 0.55%).

## Discussion

This study demonstrated that the adipose tissue areas estimated with the AZE Virtual Place software is highly reproducible and repeatable. Our study segmented abdominal SAT and VAT area estimates on CT scans via automatic planimetry software using the AZE Virtual Place software, which is substantially different from manual segmentation as done by Thaete *et al*. The SAT area estimates were more consistent than VAT area estimates. This finding was anticipated because estimates of SAT area do not require subsequent removal of blood vessels and muscle from the adipose tissue estimates. In contrast, some minor manual adjustments may be needed to remove fat-containing tissues from the bowel wall and back muscle within the VAT areas. This may introduce random operational errors. Nevertheless, this study demonstrated the high reproducibility and repeatability of AZE Virtual Place. This can reduce the overall manual work involved in abdominal adipose tissue measurements.

This study also showed that the intra-rater repeatability of measurements by a more experienced operator was better than the less experienced operator. This is because manual removal of fat-containing tissues from the bowel wall and back may introduce random operational errors. These results highlight the need for technological experience to achieve high precision.

The SAT and VAT areas were estimated from images of 10-mm reconstructed slice thickness. In contrast to the single thin-slice planimetric and the multi-slice volumetric methods, our method may benefit from the effect of voxel averaging and hence reduce the difference between SAT-to-VAT volume ratio and SAT-to-VAT area ratio^27^,^28^. Furthermore, studies on the concordance of both the planimetric and the volumetric methods with the current method are needed. Of note, the current method resulted in an average effective dose equivalent to one-eighteenth that of the average yearly effective dose received from background radiation in the US^29^.

Our study has a few limitations. First, 80% of the participants were females, and 90% of the subjects were overweighed/obese. The results suggest that estimates of body fat are reproducible and repeatable for use in treatment of this population. Although the inter- and intra- rater correlations were very high, the measurement consistency of abdominal adipose tissues using the AZE Virtual Place can vary from one institution to another. Technical variations during the post-processing of images can occur due to insufficient anatomical knowledge or on-site training time before the application. This study could not differentiate brown fat from white fat with CT numbers optimized for segmenting adipose tissue from muscle. Based on ^18^F-fluorodeoxyglucose PET (positron emission tomography)/CT studies, metabolically active brown fat has higher Hounsfield units (HUs) than inert white fat^30^,^31^. Further studies are needed to separate brown fat from white fat based on proper CT selection because it is metabolically active and predominates in lean adults.

In conclusion, this study describes the inter-rater reproducibility and intra-rater repeatability of abdominal SAT and VAT segmentation on CT images. The excellent measurement consistency of the AZE Virtual Place can evaluate adiposity changes and can facilitate studies of therapeutic outcomes.

## Methods

### Participants

This study was conducted in a single institution (Wan Fang Hospital, Taipei Medical University) and received protocol approval from the Ethics Committee of the Joint Institutional Review Board at Taipei Medical University. All methods were performed in accordance with the relevant guidelines and regulations; informed consent was obtained from all subjects.

We selected the study population from a previous study that contained 188 volunteers who met the diagnostic criteria for metabolic syndrome^32^. In the previous study, all participants were randomly assigned into one of four calorie-restriction diet groups to determine the effects of different diet regimens in patients with metabolic syndrome. This study only assessed a subset of the 188 eligible individuals because this study tested the reproducibility and repeatability of CT-based measurements of abdominal subcutaneous and visceral adipose tissues. These were unnecessary and time-consuming if all eligible individuals had post-processing segmentation. Thus, we randomly selected 20 subjects from two of the four groups (i.e. total of 40 subjects) for measuring CT reproducibility and repeatability. There were eight males and 32 females; the mean age for males was 44 (range 26–66) years and 49 (range 22–65) years for females.

All 40 subjects had one CT scan. Post-processing segmentation was only done on the SAT and VAT areas from each subject. All 40 subjects were chosen for assessment of inter-rater agreement, and 20 out of 40 subjects were randomly selected for assessment of intra-rater agreement.

### CT scans and image post-processing

**Figure 3:**
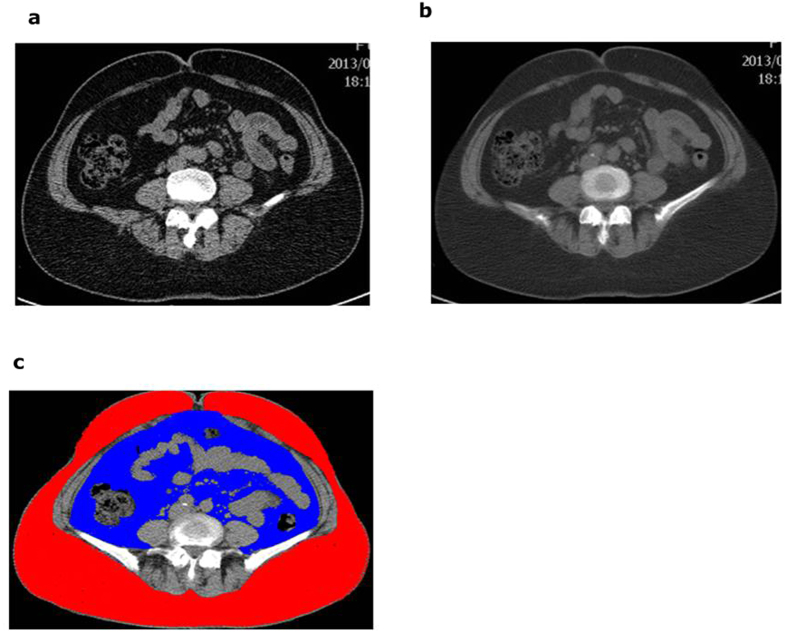
Axial views of an abdominal CT scan. An image taken through the umbilical plane with slice thickness of 2.5 mm. (**b**) A smooth image with the slice thickness of 10 mm was reconstructed from four 2.5-mm thick contiguous slices. (**c**) The SAT and VAT area estimated by AZE Virtual Place software from the reconstructed image (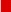: SAT, 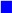: VAT).

CT scans (LightSpeed VCT; GE Healthcare, Waukesha, WI, USA) were performed with all subjects supine, a tube voltage of 120 kVp, and the tube current set to 100 mA (for BMI [body mass index] range: 25–30 kg/m^2^), 200 mA (for BMI range: >30 kg/m^2^ and ≤35 kg/m^2^), and 300 mA (for BMI > 35 kg/m^2^). The CT images of abdominal adipose tissue were obtained with the following parameters: scan FOV, large body; rotation time, 0.5 second; section thickness, 2.5 mm; and inter-slice interval, 10 mm. Eight 2.5-mm-thick original transaxial images were centered at the umbilical region and were used to reconstruct four 5.0 mm-thick images, which in turn were used to reconstruct two 10.0-mm thick images for estimating abdominal SAT and VAT areas. The SAT was defined as the extra-peritoneal fat between skin and muscles with attenuation ranging from −190 to −50 HU (Hounsfield units) on one of the constructed images at the umbilical plane^33^. The VAT was defined by intraperitoneal fat with the same density as the SAT layer (Fig. 3).

To assess the inter-rater reproducibility and intra-rater repeatability of adipose measurement with the AZE Virtual Place program, two well-trained radiologic technologists with three (Rater A) and nine (Rater B) years of professional experience on CT scans, respectively, performed the post-processing measurements. For assessing inter-rater reproducibility, each rater independently measured 40 SAT/VAT areas from 40 subjects whose SAT/VAT areas were conducted once by each rater—this resulted in two overall measurements per patient. To assess intra-rater repeatability, each rater independently measured 20 SAT/VAT areas from 20 subjects whose SAT/VAT areas were conducted twice by each rater. This resulted in four overall measurements per patient; each measurement of the same subject from each rater was done two weeks apart. The segmentation of abdominal SAT and VAT areas was determined simultaneously using automated planimetry software on a dedicated offline workstation (Virtual Place; AZE Inc., Tokyo, Japan).

The process of estimating abdominal adipose tissue area was optimized by manually removing residual fat-containing tissues such as bone marrow, bowel wall, and low-density muscle mass. We compared both SAT and VAT areas as well as fat rates. The fat rate was defined as the VAT area expressed as a percentage of the total abdominal adipose tissue area; the total abdominal adipose tissue area was calculated by adding the SAT and VAT areas.

### Statistical analyses

The measurement consistency of SAT area, VAT areas, or fat rate was determined with the CV (coefficient of variation) value, which was averaged over 40 or 20 times of inter-rater or intra-rater variability calculation, respectively. Individual inter-rater or intra-rater CV values were acquired by dividing the variance of two independent measurements by the mean of the two measurements of each subject. The overall CV was calculated by averaging the CV values, and it was expressed as the mean standard deviation. Inter-rater reproducibility and intra-rater repeatability were assessed with the Pearson product-moment correlation for determining the strength of the linear relationship between two measurements. Statistical analyses were performed using Excel; a value of p < 0.05 was considered statistically significant.

## Additional Information

**How to cite this article**: Lee, Y.-H. *et al*. Reproducibility and Repeatability of Computer Tomography-based Measurement of Abdominal Subcutaneous and Visceral Adipose Tissues. *Sci. Rep.* **7**, 40389; doi: 10.1038/srep40389 (2017).

**Publisher's note:** Springer Nature remains neutral with regard to jurisdictional claims in published maps and institutional affiliations.

## Supplementary Material

Supplementary Table 1

## Figures and Tables

**Table 1:** Age and BMI characteristics of the overall participants for assessment of inter-rater agreement.

Sex	Subjects (n = 40)	Age (years)		BMI (kg/m^2^)		Number of subjects		
		Mean	Range	Mean	Range	BMI < 25 (n = 4)	25 ≤ BMI < 30 (n = 20)	BMI ≥ 30 (n = 16)
Male	8	44	26–66	32.1	24.2–41.2	1	2	5
Female	32	49	22–65	29.5	23.5–38.1	3	18	11

BMI: body mass index.

n: number of participants.

**Table 2:** Inter-rater variation in abdominal adipose tissue area measurements.

Variables	No. of measurements	Mean	Averaged CV (±SD) (%)
SAT (cm^2^)	40	292.07	1.43 ± 1.47
VAT (cm^2^)	40	142.23	2.20 ± 2.35
Fat rate (%)	40	33.33	0.96 ± 0.96

Note: The coefficient of variance (CV) was averaged over all 40 subjects and is presented as mean CV and standard deviation.

**Table 3:** Intra-rater variation in abdominal adipose tissue area estimates.

Estimation		No. of measurements	Mean (±SD) of estimation I	Mean (±SD) of estimation II	Averaged CV (±SD) (%)
Rater A	SAT (cm^2^)	20	309.78 ± 137.05	307.14 ± 138.82	1.16 ± 1.41
	VAT (cm^2^)	20	159.13 ± 72.15	157.19 ± 70.39	2.13 ± 2.37
	Fat rate (%)	20	34.81 ± 13.32	34.76 ± 12.98	1.13 ± 1.13
Rater B	SAT (cm^2^)	20	307.09 ± 138.12	306.15 ± 137.77	0.47 ± 0.57
	VAT (cm^2^)	20	158.66 ± 71.23	157.56 ± 70.81	1.08 ± 0.97
	Fat rate (%)	20	34.99 ± 13.19	34.88 ± 13.22	0.55 ± 0.62

Note: The coefficient of variance (CV) was averaged over all 20 subjects and is presented as the mean CV and standard deviation.
