# Supplementary material for: Reproducibility and Repeatability of Computer Tomography-based Measurement of Abdominal Subcutaneous and Visceral Adipose Tissues
Source: Sci Rep. 2017 Jan 10;7:40389. doi: 10.1038/srep40389 (PMC5223165; doi:10.1038/srep40389)
Supplement: Supplementary Table 1 [file srep40389-s1.pdf]

Reproducibility and Repeatability of Computer Tomography-based Measurement of Abdominal Subcutaneous and Visceral Adipose Tissues

Yuan-Hao Lee, Hsing-Fen Hsiao, Hou-Ting Yang, Shih-Yi Huang, Wing P. Chan

Supplemental Table 1: Age and BMI characteristics of the participants for assessment of intra-rater agreement.

Sex	Subjects (n=20)	Age (years)		BMI (kg/m^2)		Number of subjects		
		Mean	Range	Mean	Range	BMI < 25 (n = 2)	25 ≤ BMI < 30 (n = 8)	BMI ≥ 30 (n = 10)
Male	5	45	27–66	34.2	28.9-41.2	0	1	4
Female	15	45	24-63	30.0	23.5–38.1	2	7	6

BMI: body mass index
n: number of participants
